# Highly Soluble Monoamino-Substituted Perylene Tetracarboxylic Dianhydrides: Synthesis, Optical and Electrochemical Properties

**DOI:** 10.3390/ijms151222642

**Published:** 2014-12-08

**Authors:** Kew-Yu Chen, Che-Wei Chang

**Affiliations:** Department of Chemical Engineering, Feng Chia University, Taichung 40724, Taiwan; E-Mail: m0111617@fcu.edu.tw

**Keywords:** perylene tetracarboxylic dianhydrides, perylene diimides, intramolecular charge transfer, solvatochromism, Lippert-Mataga equation, density functional theory calculations

## Abstract

Three dialkylamino-substituted perylene tetracarboxylic dianhydrides with different *n*-alkyl chain lengths (*n* = 6, 12 or 18), **1a**–**1c**, were synthesized under mild conditions in high yields and were characterized by ^1^H NMR, ^13^C NMR and high resolution mass spectroscopy. Their optical and electrochemical properties were measured using UV-Vis and emission spectroscopic techniques, as well as cyclic voltammetry (CV). This is the first time that the structures and the properties of monoamino-substituted perylene tetracarboxylic dianhydrides have been reported. These molecules show a deep green color in both solution and the solid state and are soluble in most organic solvents. They all show a unique charge transfer emission in the near-infrared region, and the associated peaks exhibit solvatochromism. The dipole moments of the compounds have been estimated using the Lippert-Mataga equation, and upon excitation, they show slightly larger dipole moment changes than those of corresponding perylene diimides, **2a**–**2c**. Additionally, Compounds **1a**–**1c** undergo two quasi-reversible one-electron oxidations and two quasi-reversible one-electron reductions in dichloromethane at modest potentials. Complementary density functional theory calculations performed on these chromophores are reported in order to gain more insight into their molecular structures and optical properties.

## 1. Introduction

Perylene diimides (PDIs) and perylene tetracarboxylic dianhydrides (PTCDs) have received considerable attention due to their potential applications in molecular electronic and optical devices, such as LCD color filters [1,2], molecular wires [3,4], photochromic materials [5,6], organic field-effect transistors (OFETs) [7,8,9,10,11,12], organic light-emitting diodes (OLEDs) [13,14,15,16,17], light-harvesting arrays [18,19] and organic solar cells (OSCs) [20,21,22,23,24,25,26,27,28,29]. Moreover, PDIs have been utilized as building blocks to construct supramolecular or artificial photosynthetic systems [30,31,32,33]. These organic molecules are advantageous due to their high molar absorptivities, excellent thermal and optical stabilities, reversible redox properties, high photoluminescence quantum yields and self-assembly behaviors [34,35,36,37,38,39,40,41,42,43,44,45,46,47,48]. The electronic characteristics of PDIs and PTCDs can also be fine-tuned by the substitution of a conjugated perylene core. Consequently, more and more PDIs and PTCDs with either electron-donating or electron-withdrawing groups have been reported in the literature [49,50,51,52,53,54,55,56,57,58,59,60,61,62,63].

**Scheme 1:**
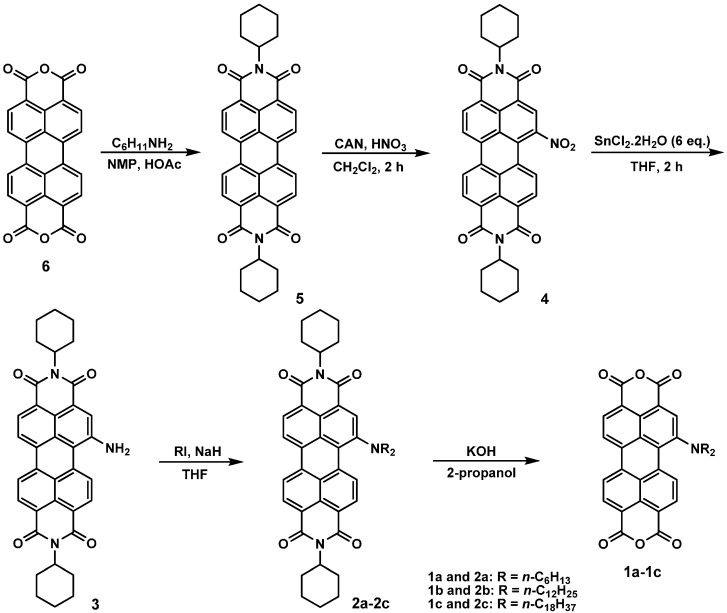
The synthetic routes for **1a**–**1c**.

PDIs and PTCDs suffer from serious problems, such as poor solubility and aggregation. A number of synthetic methods to prepare PDI and PTCD derivatives with improved solubility have been reported [53,55,59]. The synthesis of highly soluble PDI and PTCD derivatives is very important for process ability and for the preparation of their thin films to be used in optoelectronics applications, such as OFETs, OLEDs and OSCs. Soluble PDI derivatives can be obtained by introducing long and bulky substituents at the perylene core and/or at the imide nitrogen atoms, while soluble PTCDs can only be prepared by introducing substituents at the perylene core. Several diamino-substituted PTCDs based on this concept have been synthesized and studied so far [64,65]. However, to the best of our knowledge, the molecular structures, as well as the optical and electrochemical properties of monoamino-substituted PTCDs have not been reported yet. To expand the scope of highly soluble PTCD-based chromophores available for designing systems for colorful dyes and charge transport, we report here the detailed synthesis and characterization of monoamino-substituted PTCDs (**1a**–**1c**), shown in Scheme 1. Furthermore, the optical, electrochemical and complementary density functional theory calculations of the newly synthesized PTCDs are investigated.

## 2. Results and Discussion

### 2.1. Synthesis

**Figure 1:**
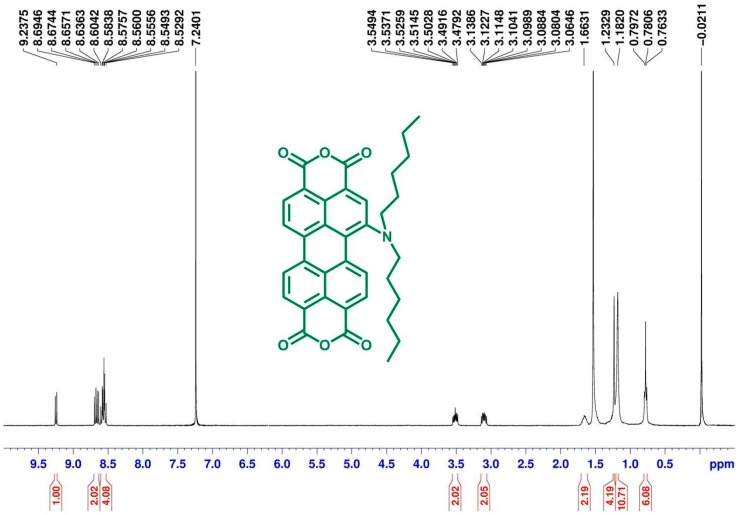
^1^H NMR (400 MHz, CDCl_3_) spectra of **1a**.

Scheme 1 depicts the chemical structures and synthetic routes of monoamino-substituted asymmetrical PTCDs (**1a**–**1c**). In brief, the synthesis of **1a**–**1c** started from an imidization of perylene dianhydride **6** and cyclohexylamine in the presence of acetic acid, followed by the mononitration of perylene diimide **5**, giving a nitro compound **4**. The reduction of 1-nitroperylene diimide (**4**) by tin (II) chloride dihydrate (SnCl_2_·2H_2_O) in refluxing THF afforded 1-aminoperylene diimide (**3**). Next, three highly soluble PDI derivatives (**2a**–**2c**) with different *N*-alkyl chain lengths (*n* = 6, 12 or 18) were prepared by the alkylation of **3** with the corresponding alkyl halides. Finally, alkylamino-substituted PDIs **2a**–**2c** were converted to the respective PTCDs via saponification to afford **1a**–**1c**. The asymmetric structure of **1a**–**1c** can be verified by the presence of seven signals (one singlet and six doublet signals) at δ 8.3–9.4 ppm in the ^1^H NMR spectrum (Figure 1), which indicates that there are seven different kinds of protons in the perylene core. Detailed synthetic procedures and product characterization are provided in the Experimental Section and Supplementary Materials (Figures S1–S10).

### 2.2. X-ray Structure

**Figure 2:**
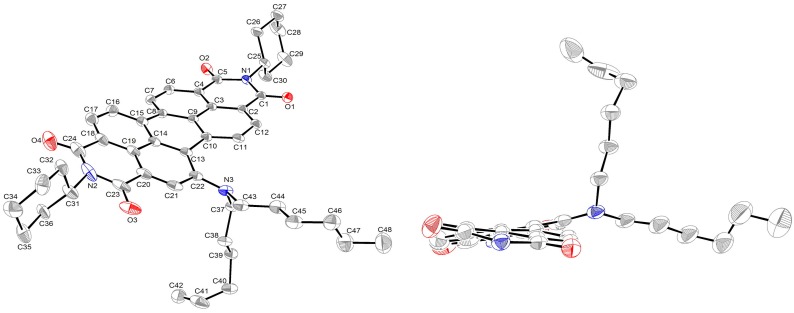
Molecular structure of **2a** (**left**) and the view along the N–N axis showing the twisted perylene backbone (**right**) (alkyl groups at imide nitrogen atoms and all hydrogen atoms are omitted for clarity). Displacement ellipsoids are drawn at the 50% probability level.

The structure of **2a** was further confirmed by single-crystal X-ray diffraction analysis (Figure 2). To the best of our knowledge, this is the first time that the X-ray structure of monosubstituted PDI has been resolved. Compound **2a** crystallizes in the triclinic space group *P*-1, with *a* = 9.8924 (6), *b* = 14.6338 (10), *c* = 15.6221 (10) Å, α = 105.098 (3)°, β = 103.386 (2)°, γ = 107.772 (3)° and *Z* = 2. The central six-membered ring of **2a** is twisted with dihedral angles of 11.6 (3)° and 14.9 (3)° associated with bay area carbon atoms C7–C8–C15–C16 and C11–C10–C13–C22, respectively, these values being smaller than those of disubstituted and tetrasubstituted PDIs [39,60]. All C–C bond lengths of the perylene moiety range between 1.358 and 1.486 Å (Table 1), which indicates the presence of π-conjugation for all C–C bonds. The longest bonds in the perylene backbone are the C8–C15 and C10–C13 bonds that connect the two naphthalene rings at a distance of 1.47 Å. The bond length is almost identical to the lengths observed for other PDIs [39,60]. These results demonstrate that the degree of conjugation of the perylene unit is similar in both unsubstituted and core-substituted PDIs.

**Table 1:** Comparison of the experimental and optimized geometric parameters of **2a** (Å and °).

Compound 2a	X-ray	DFT
Bond lengths	(Å)	(Å)
C(1)–O(1)	1.220 (3)	1.227
C(1)–N(1)	1.403 (3)	1.403
C(1)–C(2)	1.469 (3)	1.481
C(3)–C(4)	1.414 (3)	1.414
C(6)–C(7)	1.388 (3)	1.397
C(8)–C(9)	1.414 (3)	1.427
C(8)–C(15)	1.464 (3)	1.464
C(10)–C(13)	1.475 (3)	1.477
C(14)–C(19)	1.427 (3)	1.429
C(20)–C(21)	1.358 (4)	1.366
C(20)–C(23)	1.486 (4)	1.487
Bond angles	(°)	(°)
O(1)–C(1)–C(2)	122.0 (2)	121.2
C(2)–C(3)–C(4)	119.3 (2)	119.2
C(11)–C(10)–C(13)	123.4 (2)	123.3
C(13)–C(14)–C(19)	120.6 (2)	120.4
C(23)–N(2)–C(24)	123.8 (3)	123.5
C(22)–N(3)–C(37)	116.8 (2)	115.8
Torsion angles	(°)	(°)
C(7)–C(8)–C(15)–C(16)	11.6 (3)	9.4
C(11)–C(10)–C(13)–C(22)	14.9 (3)	13.4
O(1)–C(1)–C(2)–C(12)	3.4 (4)	0.7
C(19)–C(14)–C(15)–C(16)	3.0 (4)	2.1

### 2.3. Optical Properties

**Figure 3:**
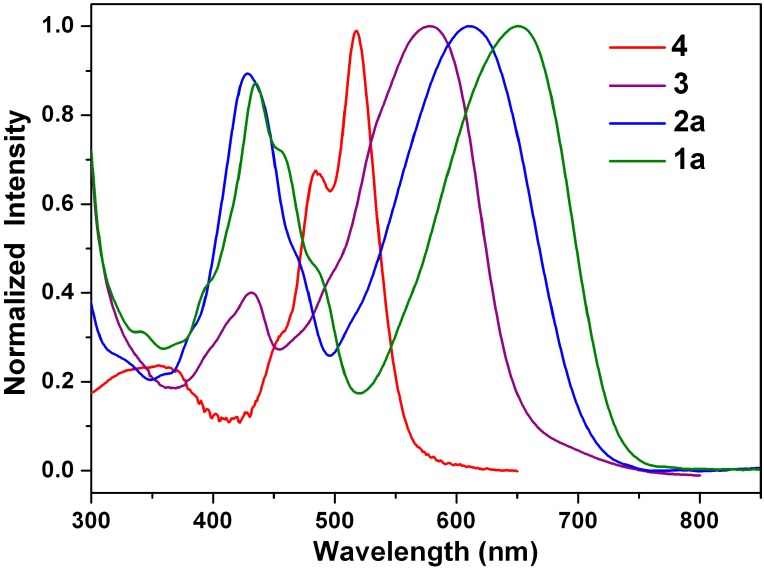
Normalized absorption spectra of **1a**, **2a**, **3** and **4** in dichloromethane.

Figure 3 shows the absorption spectra of the green dyes **1a** and **2a**, the purple dye **3** and the red dye **4** in dichloromethane. The absorption spectrum of 1-nitroperylene diimide (**4**) is almost identical with the spectrum of the non-substituted perylene diimide (**5**), but it does not show fluorescence [61]. On the other hand, the reduction of **4** to **3** switches the substituent from an electron-withdrawing nitro group to an electron-donating amino group and causes a significant red shift. The spectra of **1a**, **2a** and **3** are dominated by very broad absorption bands that cover a large part of the visible spectrum (350–750 nm). These broad bands are representative of perylene diimide (dianhydride) derivatives *N*-substituted at the bay-core positions, due to charge transfer absorption [53]. The longest wavelength absorption band of 1-(*N*,*N*-dihexylamino)perylene diimide (**2a**: 610 nm) is red-shifted relative to that of 1-aminoperylene diimide (**3**: 578 nm), but it is blue-shifted relative to that of 1-(*N*,*N*-dihexylamino)perylene tetracarboxylic dianhydride (**1a**: 650 nm). It appears that the inductive effect of the alkyl groups in **1a** and **2a** causes an additional red shift. Interestingly, the longest wavelength absorption band of **1a** is 40-nm red-shifted relative to that of **2a**; the decrease in the energy band gap is attributed to a great decrease in the LUMO energy level (*vide infra*).

**Figure 4:**
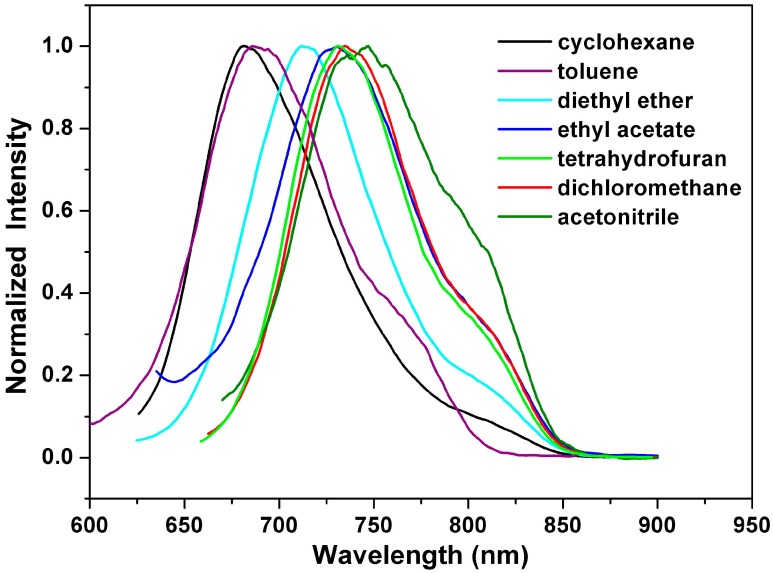
Normalized emission spectra of **1a** in various solvents.

Figure 4 depicts the emission spectra of **1a** in solvents of varying polarity, where those of **1b** and **1c** can be found in the Supplementary Materials (Figures S11 and S12). Unlike the small shift in absorption spectra, the fluorescence spectra of **1a**–**1c** are largely red-shifted if there is any increase of the solvent polarity, which indicates strong intramolecular charge transfer characteristics for the excited states of **1a**–**1c** (Table 2). Using the fluorescence solvatochromic shift method [66], we measured the stabilization of the excited-states of **1a**–**1c** and compared these results to those of **2a**–**2c**. The change of magnitudes for dipole moments between ground and excited states, *i.e.*, $\Delta \text{μ}=|\vec{{\text{μ}}_{e}}-\vec{{\text{μ}}_{g}}|$, can be estimated by the Lippert–Mataga Equation (1) and expressed as:

(1) $${\bar{\upsilon }}_{a}-{\bar{\upsilon }}_{f}=\frac{2}{hc}{\left({\text{μ}}_{e}-{\text{μ}}_{g}\right)}^{2}{a_{0}}^{-3}\text{Δ}f+const.$$

where *h* is the Planck constant, *c* is the speed of light, $a_{0}$ denotes the cavity radius in which the solute resides, ${\bar{\upsilon }}_{a}-{\bar{\upsilon }}_{f}$ is the Stokes shift of the absorption and emission peak maximum, and Δf is the orientation polarizability as shown in Equation (2).

(2) $$\text{Δ}f=f\left(\text{ε}\right)-f\left(n^{2}\right)=\frac{\text{ε}-1}{2\text{ε}+1}-\frac{n^{2}-1}{2n^{2}+1}$$

**Figure 5:**
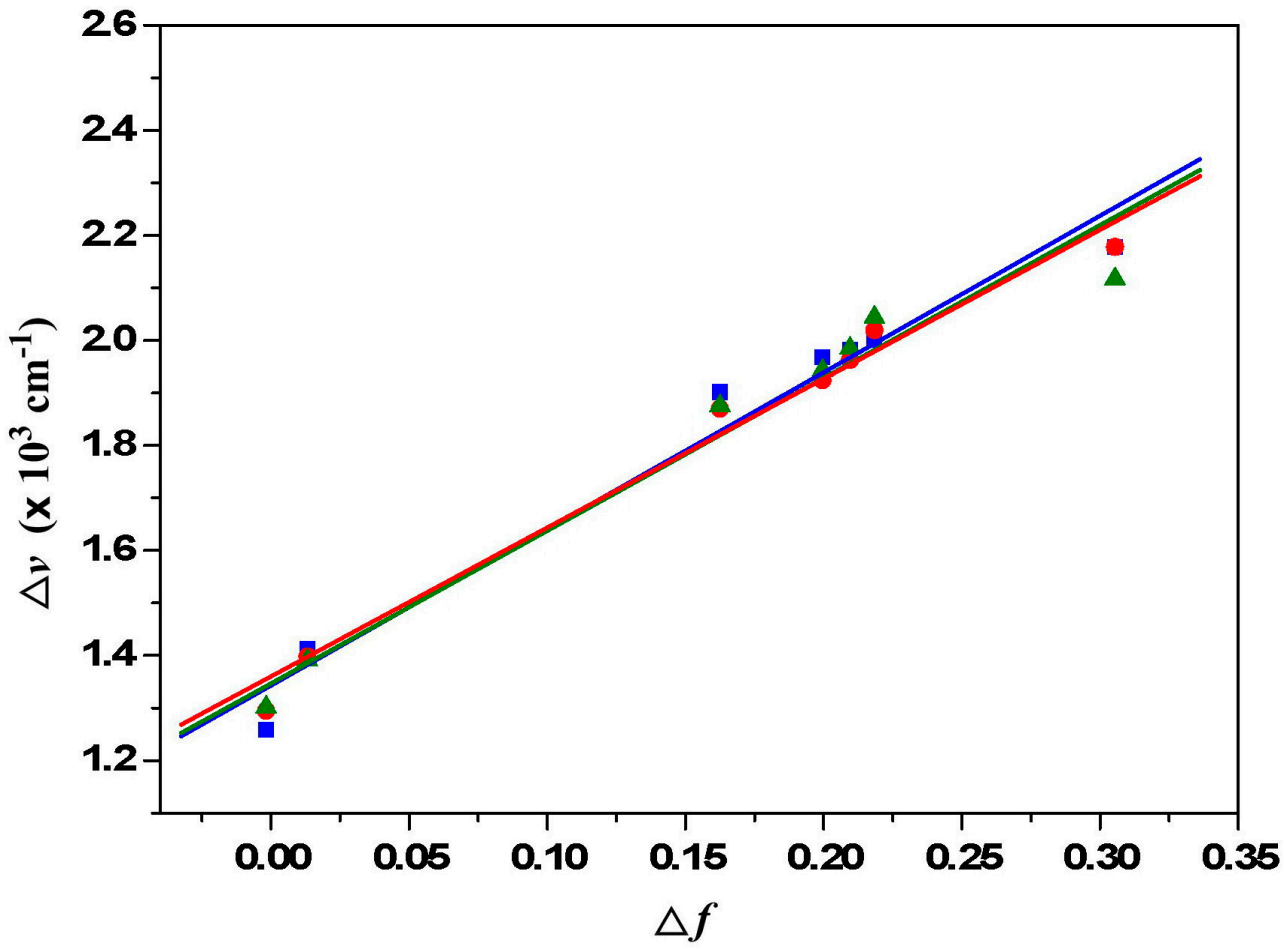
Lippert-Mataga plots for **1a** (red line and red symbols), **1b** (green line and green symbols) and **1c** (blue line and blue symbols). The solvents from left to right are (1) cyclohexane, (2) toluene, (3) diethyl ether, (4) ethyl acetate, (5) tetrahydrofuran, (6) dichloromethane and (7) acetonitrile.

The plot of the Stokes shift ${\bar{\upsilon }}_{a}-{\bar{\upsilon }}_{f}$ as a function of Δf is sufficiently linear for **1a**–**1c** (Figure 5). Accordingly, $\Delta \text{μ}=|\vec{{\text{μ}}_{e}}-\vec{{\text{μ}}_{g}}|$ values can be calculated as 9.5 D, 11.9 D and 13.0 D for **1a**–**1c**. These values indicate that dialkylamino-substituted PTCDs (**1a**–**1c**) have slightly larger dipole moment changes than those of corresponding dialkylamino-substituted PDIs (9.0 D, 11.7 D and 12.8 D for **2a**–**2c**).

**Table 2:** Summary of optical absorption and emission properties of **1a**–**1c** in various solvents.

1a/1b/1c	λ_abs_ (nm) ^a^	λ_em_ (nm) ^a^	Stokes Shift (nm)	Φ ^b^ × 10^3^
cyclohexane	623/624/624	678/677/679	55/53/55	1.21/1.93/7.83
toluene	626/627/627	686/686/687	60/59/60	1.01/1.15/1.38
diethyl ether	624/626/625	708/709/708	84/83/83	1.03/1.19/1.70
ethyl acetate	636/637/637	727/726/727	91/89/90	0.90/0.81/0.96
tetrahydrofuran	638/639/639	731/732/731	93/93/92	0.48/0.55/0.62
dichloromethane	640/640/639	734/735/735	94/95/96	0.29/0.39/0.59
acetonitrile	641/641/642	745/745/743	104/104/101	0.16/0.24/0.33

^a^ Measured at 2 × 10^−5^ M; ^b^ determined with *N*,*N*′-dioctyl-3,4,9,10-perylenedicarboximide as the reference [31].

### 2.4. Electrochemical Properties

**Figure 6:**
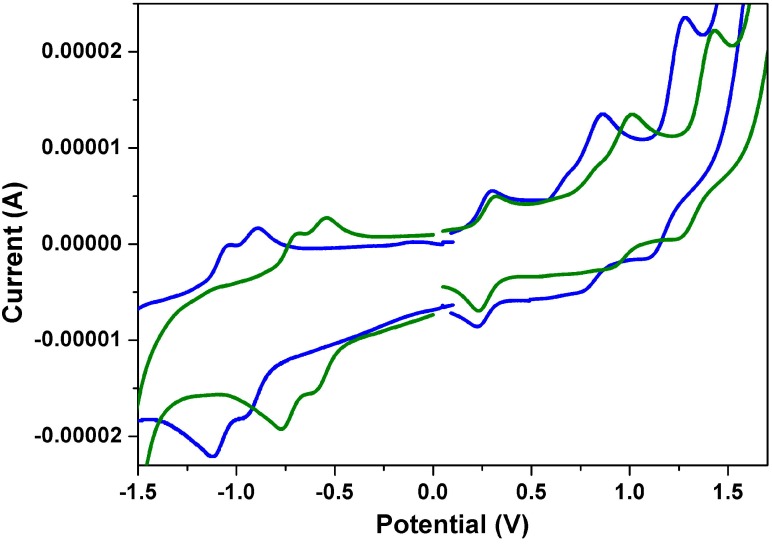
The cyclic voltammograms of **1a** (green line) and **2a** (blue line) measured in dichloromethane solution with ferrocenium/ferrocene as an internal standard, at 200 mV/s.

Figure 6 shows the cyclic voltammograms of **1a** and **2a**. Both undergo two quasi-reversible one-electron oxidations and two quasi-reversible one-electron reductions in dichloromethane at modest potentials. One can clearly see that both the first oxidation and the first reduction potentials of **1a** are larger than those of **2a**; this can be explained by the fact that the anhydride group is a stronger electron-withdrawing group than the imide group. Table 3 summarizes the redox potentials and the HOMO and LUMO energy levels estimated from cyclic voltammetry (CV) for **1a**–**1c** and **2a**–**2c**. The HOMO/LUMO energy levels of **1a**, **1b**, **1c**, **2a**, **2b** and **2c** are estimated to be −5.67/−3.68, −5.65/−3.67, −5.66/−3.69, −5.46/−3.35, −5.47/−3.36 and −5.45/−3.34 eV, respectively. The HOMO-LUMO energy gaps of **1a**–**1c** are found to be virtually the same, which indicates that different *N*-alkyl chain lengths do not significantly affect the band gap energies.

**Table 3:** Summary of half-wave redox potentials, HOMO and LUMO energy levels for **1a**–**1c** and **2a**–**2c**.

Compound	*E*^+^_1/2_^a^	*E*^2+^_1/2_^a^	*E*^−^_1/2_^a^	*E*^2−^_1/2_^a^	HOMO ^b^	LUMO ^b^
**1a**	1.05	1.32	−0.65	−0.79	−5.67	−3.68
**1b**	1.03	1.31	−0.66	−0.80	−5.65	−3.67
**1c**	1.04	1.30	−0.64	−0.78	−5.66	−3.69
**2a**	0.84	1.11	−0.97	−1.09	−5.46	−3.35
**2b**	0.85	1.13	−0.95	−1.10	−5.47	−3.36
**2c**	0.83	1.12	−0.96	−1.08	−5.45	−3.34

^a^ Measured in a solution of 0.1 M tetrabutylammonium hexafluorophosphate (TBAPF_6_) in dichloromethane *versus* saturated calomel electrode (in V); ^b^ calculated from *E*_HOMO_ = −4.88 − (*E*_oxd_ − *E*_Fc/Fc+_), *E*_LUMO_ = *E*_HOMO_ + *E*_g_.

### 2.5. Quantum Chemistry Computation

**Figure 7:**
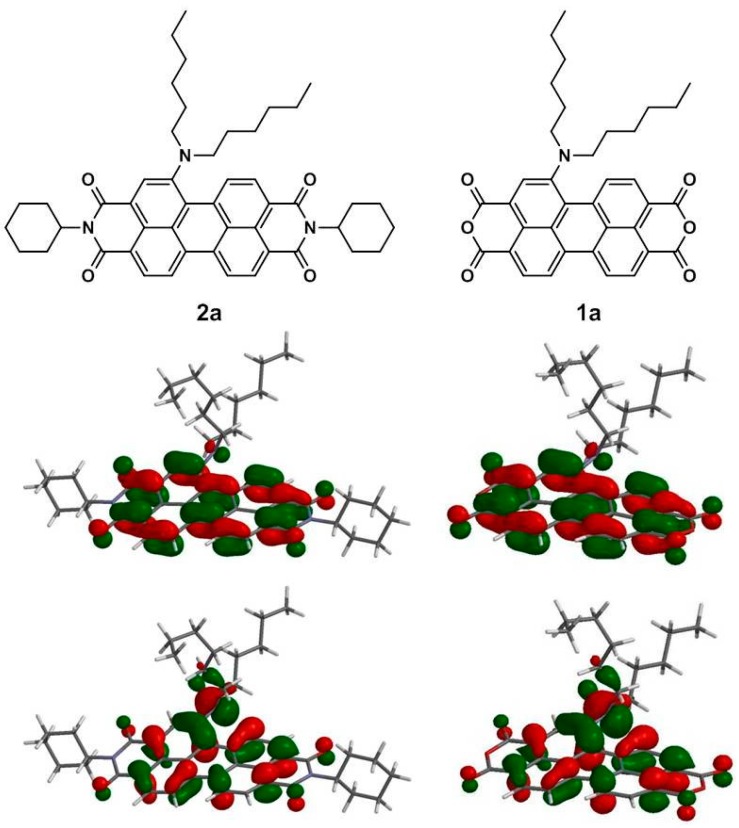
Computed frontier orbitals of **1a** and **2a**. The upper graphs are the LUMOs, and the lower ones are the HOMOs.

To gain deeper insight into the molecular structures and electronic properties of **1a**–**1c** and **2a**–**2c**, quantum chemical calculations were performed using the density functional theory (DFT) at the B3LYP/6-31G** level. Figure 7 depicts the highest occupied molecular orbitals (HOMOs) and the lowest unoccupied molecular orbitals (LUMOs) of **1a** and **2a**. The HOMO of **1a** (**2a**) is delocalized mainly on the amino group and the perylene core, while the LUMO is extended from the central perylene core to the dianhydride (diimide) groups. The calculated and experimental parameters for **1a**–**1c** and **2a**–**2c** are summarized in Table 4. It is apparent that both the HOMO and LUMO energy levels of **1a**–**1c** are lower than those of **2a**–**2c** and are in good agreement with the experimental data.

**Figure 8:**
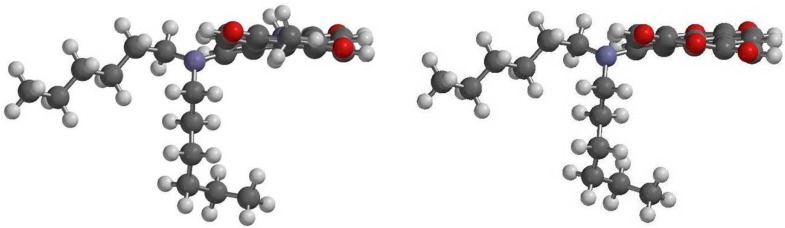
DFT (B3LYP/6-31G**) geometry-optimized structures of **1a** (**right**) and **2a** (**left**) shown with the view along the long perylene axis. For computational purposes, methyl groups replace the cyclohexyl groups at the imide positions.

DFT calculations also show that the ground-state geometries of the perylene core have different core twist angles (Figure 8), *i.e.*, approximate dihedral angles between the two naphthalene subunits attached to the central benzene ring; these are ~8.57° and ~12.42° for **1a**, ~8.59° and ~12.45° for **1b**, ~8.61° and ~12.44° for **1c**, ~9.40° and ~13.43° for **2a**, ~9.42° and ~13.45° for **2b** and ~9.45° and ~13.49° for **2c** (Table 4); and all are larger than those of **5** (~0.00°). As a whole, the core twist angles of dialkylamino-substituted PTCDs (**1a**–**1c**) are slightly smaller than those of corresponding PDIs (**2a**–**2c**).

**Table 4:** Calculated and experimental parameters for **1a**–**1c** and **2a**–**2c**.

Compound	HOMO ^a^	LUMO ^a^	*E*_g_^a^	*E*_g_^b^	*μ*_g_^c^	*μ*_e_^d^	Twisting Angle (°)
**1a**	−5.81	−3.64	2.17	1.99	4.1	13.6	8.57, 12.42
**1b**	−5.80	−3.64	2.16	1.98	4.3	16.2	8.59, 12.45
**1c**	−5.80	−3.64	2.16	1.97	4.6	17.6	8.61, 12.47
**2a**	−5.48	−3.19	2.29	2.11	3.5	12.5	9.40, 13.43
**2b**	−5.48	−3.19	2.29	2.11	3.6	15.3	9.42, 13.45
**2c**	−5.47	−3.19	2.28	2.11	3.8	16.6	9.45, 13.49

^a^ Calculated by DFT/B3LYP (in eV); ^b^ at absorption maxima (*E*_g_ = 1240/λ_max_, in eV); ^c^ ground-state dipole moment (calculated by DFT/B3LYP, in Debye); ^d^ excited-state dipole moment (in Debye).

### 2.6. Stacking Behaviors of Dyes in Solution and Solid State

**Figure 9:**
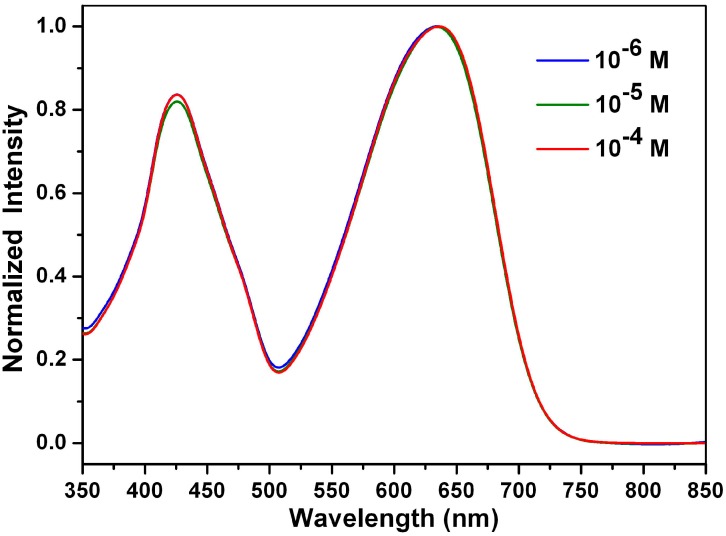
Normalized absorption spectra of **1a** at different concentrations in ethyl acetate.

The stacking behaviors of **1a**–**1c** and **3** in ethyl acetate were investigated by concentration-dependent UV-Vis measurements (from 10^−4^ to 10^−6^ M). The absorption spectra of **1a** at different concentrations in ethyl acetate are shown in Figure 9; the absorption spectra of **1b**, **1c** and **3** at different concentrations in ethyl acetate are shown in the Supplementary Materials (Figures S13–S15). At high concentrations, a clear red shift was observed for 1-aminoperylene bisimide **3**, indicating the formation of J-type aggregates [51]. However, compounds **1a**–**1c** show no significant red shift at high concentrations of ethyl acetate, which can be explained by the fact that the long alkyl side chains of **1a**–**1c** decrease the π–π interactions.

**Figure 10:**
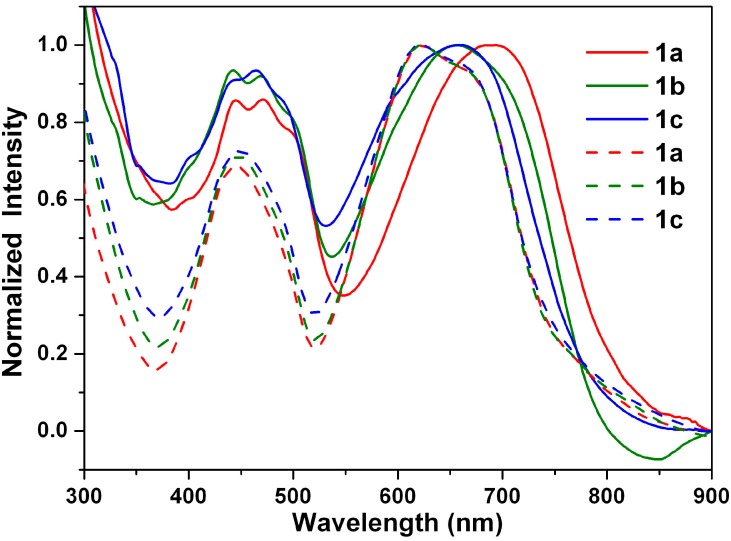
Normalized absorption spectra of **1a**–**1c** in cyclohexane solution (dashed line) and in neat film (solid line).

Figure 10 shows the absorption spectra recorded for the thin drop-cast films of **1a**–**1c**. The shapes of the absorption spectra of **1a**–**1c** in the solid state and in solution show significant differences, in view of the wavelength range and peak positions. The absorption spectra of all of the drop-cast film chromophores were broadened, as well as red-shifted compared to their respective spectra in cyclohexane solution, which indicates aggregation. This spectral change can be mainly attributed to intermolecular π–π interactions in the solid state [67].

## 3. Experimental Section

### 3.1. General

The starting materials, such as perylene-3,4,9,10-tetracarboxylic dianhydride, acetic acid, cyclohexylamine, cerium(IV) ammonium nitrate (CAN), 1-methyl-2-pyrrolidinone (NMP), tetrahydrofuran (THF), tin (II) chloride dihydrate (SnCl_2_·2H_2_O), KOH and 2-propanol, were purchased from Merck (Whitehouse Station, NJ, USA), ACROS (Pittsburgh, PA, USA) and Sigma-Aldrich (St. Louis, MO, USA). Solvents were distilled freshly according to the standard procedure. Column chromatography was performed using silica gel Merck Kieselgel *si* 60 (40–63 mesh). ^1^H or ^13^C NMR spectra were recorded in CDCl_3_ on a Bruker 400 or 500 MHz instrument (Palo Alto, CA, USA). Mass spectra were recorded on a VG70-250S mass spectrometer (Tokyo, Japan). The absorption and emission spectra were measured using a Jasco V-570 UV-Vis spectrophotometer (Tokyo, Japan) and a Hitachi F-7000 fluorescence spectrophotometer (Tokyo, Japan), respectively. The Gaussian 03 program (Pittsburgh, PA, USA) was used to perform the *ab initio* calculation on the molecular structure. Geometry optimizations for Compounds **1a**–**1c** and **2a**–**2c** were carried out with the 6-31G** basis set to the B3LYP functional. Vibrational frequencies were also performed to check whether the optimized geometrical structures for all compounds were at energy minima, transition states or higher order saddle points.

### 3.2. Synthesis

#### 3.2.1. Synthesis of 1-Nitroperylene Diimide (**4**)

Compound **5** (1.8 mmol), cerium (IV) ammonium nitrate (1.2 g, 2.2 mmol), nitric acid (2.0 g, 31.7 mmol) and dichloromethane (150 mL) were stirred at 25 °C under N_2_ for 2 h. The mixture was neutralized with 10% KOH and extracted with CH_2_Cl_2_. After the solvent was removed, the crude product was purified by silica gel column chromatography with eluent CH_2_Cl_2_ to afford **4** in a 95% yield. Characterization data: ^1^H NMR (500 MHz, CDCl_3_) δ 8.74 (1H, d, *J* = 7.6 Hz), 8.62–8.69 (4H, m), 8.55 (1H, d, *J* = 8.5 Hz), 8.18 (1H, d, *J* = 7.6 Hz), 5.00 (2H, m), 2.54 (4H, m), 1.91 (4H, m), 1.76 (6H, m), 1.47 (4H, m), 1.34 (2H, m); MS (FAB): *m*/*z* (relative intensity) 600 [M + H^+^, 100]; HRMS calcd. for C_36_H_30_O_6_N_3_ 600.2135, found 600.2141.

#### 3.2.2. Synthesis of 1-Aminoperylene Diimide (**3**)

Tin chloride dihydrate (2.5 g, 10.2 mmol) and **4** (1.0 g, 1.7 mmol) were suspended in THF (50 mL) and stirred 20 min. The solvent was refluxed at 80 °C with stirring for 2 h. THF was removed at the rotary evaporator, and the residue was dissolved in ethyl acetate and washed with 10% sodium hydrate solution and brine. The organic layer was dried over anhydrous MgSO_4_, and the filtrate was concentrated under reduced pressure. The crude product was purified by silica gel column chromatography with eluent ethyl acetate/*n*-hexane (2/3) to afford **3** in an 80% yield. Characterization data: **3**: ^1^H NMR (400 MHz, CDCl_3_) δ 8.62 (1H, d, *J* = 8.0 Hz), 8.45 (1H, d, *J* = 7.6 Hz), 8.38 (1H, d, *J* = 8.0 Hz), 8.25 (1H, d, *J* = 7.6 Hz), 8.18 (1H, d, *J* = 8.0 Hz), 8.10 (1H, d, *J* = 8.0 Hz), 7.98 (1H, s), 5.03 (2H, s), 4.99 (2H, m), 2.55 (4H, m), 1.91 (4H, m), 1.74 (6H, m), 1.46–1.40 (6H, m); MS (FAB): *m*/*z* (relative intensity) 570 [M + H^+^, 100]; HRMS calcd. for C_36_H_32_O_4_N_3_ 570.2393, found 570.2396.

#### 3.2.3. General Procedure for Alkylation (**2a**–**2c**)

A mixture of a solution of **3** (400 mg, 0.70 mmol), sodium hydride (97%, 100 mg, 4.00 mmol) and dry THF (50 mL) was stirred at 0 °C under N_2_ for 30 min. Alkyl iodide (1.60 mmol) was then added, and the resulting mixture was stirred for 2 h. The resulting mixture was diluted with 15 mL of water and extracted with CH_2_Cl_2_. The crude product was purified by silica gel column chromatography with eluent ethyl acetate/*n*-hexane (1/2) to afford **2a** (**2b** or **2c**) in an 85% yield. Characterization data: **2a**: ^1^H NMR (400 MHz, CDCl_3_) δ 9.32 (d, *J* = 8.0 Hz, 1H), 8.49–8.53 (m, 2H), 8.48 (s, 1H), 8.31–8.39 (m, 3H), 5.02 (m, 2H), 3.40 (m, 2H), 3.06 (m, 2H), 2.55 (m, 4H), 1.90 (m, 4H), 1.62–1.79 (m, 8H), 1.46 (m, 4H), 1.16–1.30 (m, 16H), 0.77 (t, *J* = 6.4 Hz, 6H); ^13^C NMR (100 MHz, CDCl_3_) δ 164.07, 164.06, 163.99, 163.86, 150.56, 135.47, 134.63, 133.53, 131.41, 130.61, 129.11, 128.95, 128.10, 126.98, 126.94, 126.93, 124.80, 124.03, 123.44, 123.19, 122.76, 122.11, 121.30, 121.19, 54.04, 53.82, 52.55, 31.44, 29.67, 29.18, 29.11, 27.47, 26.89, 26.59, 25.51, 22.52, 13.89; MS (FAB): *m*/*z* (relative intensity) 738 [M + H^+^, 100]; HRMS calcd. for C_48_H_56_O_4_N_3_ 738.4271, found 738.4277. Selected data for **2b**: ^1^H NMR (400 MHz, CDCl_3_) δ 9.29 (d, *J* = 8.0 Hz, 1H), 8.47–8.52 (m, 2H), 8.46 (s, 1H), 8.29–8.43 (m, 3H), 5.01 (m, 2H), 3.38 (m, 2H), 3.05 (m, 2H), 2.56(m, 4H), 1.90 (m, 4H), 1.60–1.77 (m, 6H), 1.46 (m, 6H), 1.11–1.20 (m, 40H), 0.82 (t, *J* = 6.7 Hz, 6H); ^13^C NMR (100 MHz, CDCl_3_) δ 164.05, 164.03, 163.97, 163.82, 150.56, 135.44, 134.60, 133.49, 131.40, 130.57, 129.09, 128.93, 128.05, 126.95, 126.91, 126.89, 124.77, 124.01, 123.42, 123.18, 122.75, 122.00, 121.27, 121.14, 54.04, 53.81, 52.49, 31.86, 29.68, 29.56, 29.50, 29.28, 29.19, 29.11, 27.50, 27.21, 26.60, 25.51, 22.64, 14.07; MS (FAB): *m*/*z* (relative intensity) 906 [M + H^+^, 100]; HRMS calcd. for C_60_H_80_O_4_N_3_ 906.6149, found 906.6141. Selected data for **2c**: ^1^H NMR (400 MHz, CDCl_3_) δ 9.37 (d, *J* = 8.0 Hz, 1H), 8.53–8.56 (m, 2H), 8.48 (s, 1H), 8.38–8.43 (m, 3H), 5.03 (m, 2H), 3.44 (m, 2H), 3.07 (m, 2H), 2.56 (m, 4H), 1.91 (m, 4H), 1.63–1.73 (m, 6H), 1.47 (m, 6H), 1.12–1.20 (m, 64H), 0.85 (t, *J* = 6.7 Hz, 6H); ^13^C NMR (100 MHz, CDCl_3_) δ 164.15, 164.09, 164.05, 163.88, 150.54, 135.56, 134.75, 133.63, 131.51, 130.70, 129.19, 129.04, 128.17, 127.03, 124.87, 124.06, 123.50, 123.46, 123.44, 123.22, 122.82, 122.22, 121.34, 121.23, 54.03, 53.82, 52.61, 31.91, 29.67, 29.64, 29.58, 29.51, 29.34, 29.27, 29.19, 29.12, 27.54, 27.22, 26.59, 25.51, 22.67, 14.09; MS (FAB): *m*/*z* (relative intensity) 1074 [M + H^+^, 100]; HRMS calcd. for C_72_H_104_O_4_N_3_ 1074.8027, found 1074.8019.

#### 3.2.4. General Procedure for Saponification (**1a**–**1c**)

**2a** (**2b** or **2c**, 0.27 mmol) was taken in 2-propanol (30 mL), and subsequently, KOH (1.9 g, 33.8 mmol) was added. The reaction mixture was stirred under N_2_ at reflux for 4 h. After being cooled to room temperature, the reaction mixture was poured into acetic acid (50 mL) and stirred overnight. The resulting green precipitate was collected by filtration, washed with water and methanol and dried. The crude product was purified by silica gel column chromatography with eluent CH_2_Cl_2_ to afford **1a** (**1b** or **1c**) in a 75% yield. Characterization data: **1a**: ^1^H NMR (400 MHz, CDCl_3_) δ 9.25 (d, *J* = 8.0 Hz, 1H), 8.60–8.69 (m, 2H), 8.52–8.58 (m, 4H), 3.54 (m, 2H), 3.13 (m, 2H), 1.18–1.63 (m, 16H), 0.87 (t, *J* = 6.4 Hz, 6H); MS (FAB): *m*/*z* (relative intensity) 576 [M + H^+^, 100]; HRMS calcd. for C_36_H_34_O_6_N 576.2386, found 576.2380. Selected data for **1b**: ^1^H NMR (400 MHz, CDCl_3_) δ 9.22 (d, *J* = 8.4 Hz, 1H), 8.59–8.67 (m, 2H), 8.51–8.57 (m, 4H), 3.54 (m, 2H), 3.13 (m, 2H), 1.13–1.65 (m, 40H), 0.87 (t, *J* = 5.2 Hz, 6H); ^13^C NMR (100 MHz, CDCl_3_) δ 164.41, 160.25, 159.97, 159.92, 151.13, 136.90, 136.12, 134.59, 133.83, 133.06, 131.82, 130.00, 129.70, 128.99, 127.69, 126.72, 124.75, 123.73, 122.09, 121.96, 119.55, 119.22, 118.60, 116.82, 52.84, 31.87, 29.70, 29.56, 29.49, 29.46, 29.29, 29.22, 27.78, 27.12, 22.66, 14.09; MS (FAB): *m*/*z* (relative intensity) 744 [M + H^+^, 100]; HRMS calcd. for C_48_H_58_O_6_N 744.4264, found 744.4272. Selected data for **1c**: ^1^H NMR (400 MHz, CDCl_3_) δ 9.22 (d, *J* = 8.4 Hz, 1H), 8.62–8.67 (m, 2H), 8.51–8.58 (m, 4H), 3.54 (m, 2H), 3.13 (m, 2H), 1.13–1.68 (m, 64H), 0.86 (t, *J* = 6.6 Hz, 6H); ^13^C NMR (100 MHz, CDCl_3_) δ 160.41, 160.22, 159.95, 159.90, 151.13, 136.90, 136.12, 134.59, 133.83, 133.05, 131.82, 129.99, 129.70, 128.99, 127.69, 126.72, 124.74, 123.73, 122.09, 121.96, 119.55, 119.22, 118.60, 116.82, 52.84, 31.92, 29.68, 29.57, 29.51, 29.47, 29.35, 29.22, 27.78, 27.13, 22.68, 14.11; MS (FAB): *m*/*z* (relative intensity) 912 [M + H^+^, 100]; HRMS calcd. for C_60_H_82_O_6_N 912.6142, found 912.6150.

### 3.3. Crystal Structural Determination

A single crystal of **2a** with dimensions of 0.48 mm × 0.12 mm × 0.03 mm was selected. The lattice constants and diffraction intensities were measured with a Bruker Smart 1000 CCD area detector radiation (λ = 0.71073 Å) at 150(2) K. An *ω*-2*θ* scan mode was used for data collection in the range of 2.22 ≤ *θ* ≤ 26.507. A total of 32,126 reflections were collected, and 8084 were independent (*R*_int_ = 0.0626), of which 4733 were considered to be observed with *I* > 2*σ* (*I*) and used in the succeeding refinement. The structure was solved by direct methods with SHELXS-97 [68] and refined on *F*^2^ by a full-matrix least-squares procedure with Bruker SHELXL-97 packing [69]. All non-hydrogen atoms were refined with anisotropic thermal parameters. The hydrogen atoms refined with riding model position parameters isotropically were located from the difference Fourier map and added theoretically. At the final cycle of refinement, *R* = 0.0644 and *wR* = 0.1601 (*w* = 1/[*σ*^2^(*F_o_*^2^) + (0.0871*P*)^2^ + 0.8402*P*], where *P* = (*F_o_*^2^ + 2*F_c_*^2^)/3), *S* = 1.047, (Δ/*σ*)_max_ = 0.001, (Δ/*ρ*)_max_ = 0.337 and (Δ/*ρ*)_min_ = −0.375 e/Å^3^). Crystallographic data for Compound **2a** have been deposited with the Cambridge Crystallographic Data Center as supplementary publication number CCDC 1030664. Copies of this information can be obtained free of charge from the Director, CCDC, 12 Union Road, Cambridge CB2 1EZ, U.K. (Fax: +44 1223 336 033; E-mail: deposit@ccdc.cam.ac.uk).

## 4. Conclusions

We have successfully synthesized three monoamino-substituted asymmetrical perylene tetracarboxylic dianhydrides with different *n*-alkyl chain lengths (**1a**–**1c**). These molecules show an intense green color in both solution and the solid state and are soluble in most organic solvents and even in nonpolar solvents, such as hexane. They show a unique charge transfer emission in the near-infrared region, of which the peak wavelengths exhibit strong solvatochromism. Upon excitation, they show slightly larger dipole moment changes than those of corresponding perylene diimides **2a**–**2c**; the dipole moments of these compounds have been estimated using density functional theory calculations and the Lippert-Mataga equation. Furthermore, they undergo two quasi-reversible one-electron oxidations and two quasi-reversible one-electron reductions in dichloromethane at modest potentials. Research on their applications to dye-sensitized solar cells (DSSCs) is currently in progress.

## Supplementary Materials

Supplementary figures can be found at http://www.mdpi.com/1422-0067/15/12/22642/s1.

## References

[1] Choi J., Lee W., Sakong C., Yuk S.B., Park J.S., Kim J.P. (2012). Facile synthesis and characterization of novel coronene chromophores and their application to LCD color filters. Dyes Pigment..

[2] Sakong C., Kim Y.D., Choi J.H., Yoon C., Kim J.P. (2011). The synthesis of thermally-stable red dyes for LCD color filters and analysis of their aggregation and spectral properties. Dyes Pigment..

[3] Weiss E.A., Ahrens M.J., Sinks L.E., Gusev A.V., Ratner M.A., Wasielewski M.R. (2004). Making a molecular wire: Charge and spin transport through para-phenylene oligomers. J. Am. Chem. Soc..

[4] Wilson T.M., Tauber M.J., Wasielewski M.R. (2009). Toward an *n*-type molecular wire: Electron hopping within linearly linked perylenediimide oligomers. J. Am. Chem. Soc..

[5] Berberich M., Krause A.M., Orlandi M., Scandola F., Würthner F. (2008). Toward fluorescent memories with nondestructive readout: Photoswitching of fluorescence by intramolecular electron transfer in a diaryl ethene-perylene bisimide photochromic system. Angew. Chem. Int. Ed..

[6] Tan W., Li X., Zhang J., Tian H. (2011). A photochromic diarylethene dyad based on perylene diimide. Dyes Pigment..

[7] Jones B.A., Ahrens M.J., Yoon M.H., Facchetti A., Marks T.J., Wasielewski M.R. (2004). High-mobility air-stable *n*-type semiconductors with processing versatility: Dicyanoperylene-3,4:9,10-bis(dicarboximides). Angew. Chem. Int. Ed..

[8] Kim F.S., Guo X., Watson M.D., Jenekhe S.A. (2009). High-mobility ambipolar transistors and high-gain inverters from a donor-acceptor copolymer semiconductor. Adv. Mater..

[9] Würthner F., Stolte M. (2011). Naphthalene and perylene diimides for organic transistors. Chem. Commun..

[10] Reghu R.R., Bisoyi H.K., Grazulevicius J.V., Anjukandi P., Gaidelis V., Jankauskas V. (2011). Air stable electron-transporting and ambipolar bay substituted perylene bisimides. J. Mater. Chem..

[11] Zaumseil J., Sirringhaus H. (2007). Electron and ambipolar transport in organic field-effect transistors. Chem. Rev..

[12] Locklin J., Li D., Mannsfeld S.C.B., Borkent E.J., Meng H., Advincula R., Bao Z. (2005). Organic thin film transistors based on cyclohexyl-substituted organic semiconductors. Chem. Mater..

[13] Ventura B., Langhals H., Böck B., Flamigni L. (2012). Phosphorescent perylene imides. Chem. Commun..

[14] Matsui M., Wang M., Funabiki K., Hayakawa Y., Kitaguchi T. (2007). Properties of novel perylene-3,4:9,10-tetracarboxidiimide-centred dendrimers and their application as emitters in organic electroluminescence devices. Dyes Pigment..

[15] Damaceanu M.-D., Constantin C.-P., Bruma N., Pinteala M. (2013). Tuning of the color of the emitted light from new polyperyleneimides containing oxadiazole and siloxane moieties. Dyes Pigment..

[16] Lucenti E., Botta C., Cariati E., Righetto S., Scarpellini M., Tordin E., Ugo R. (2013). New organic-inorganic hybrid materials based on perylene diimide-polyhedral oligomeric silsesquioxane dyes with reduced quenching of the emission in the solid state. Dyes Pigment..

[17] Pan J., Zhu W., Li S., Zeng W., Cao Y., Tian H. (2005). Dendron-functionalized perylene diimides with carrier-transporting ability for red luminescent materials. Polymer.

[18] Li X., Sinks L.E., Rybtchinski B., Wasielewski M.R. (2004). Ultrafast aggregate-to-aggregate energy transfer within self-assembled light-harvesting columns of zinc phthalocyanine tetrakis(perylenediimide). J. Am. Chem. Soc..

[19] Rybtchinski B., Sinks L.E., Wasielewski M.R. (2004). Combining light-harvesting and charge separation in a self-assembled artificial photosynthetic system based on perylenediimide chromophores. J. Am. Chem. Soc..

[20] Kozma E., Kotowski D., Catellani M., Luzzati S., Famulari A., Bertini F. (2013). Synthesis and characterization of new electron acceptor perylene diimide molecules for photovoltaic applications. Dyes Pigment..

[21] Li J., Dierschke F., Wu J., Grimsdale A.C., Müllen K. (2006). Poly(2,7-carbazole) and perylene tetracarboxydiimide: A promising donor/acceptor pair for polymer solar cells. J. Mater. Chem..

[22] Dinçalp H., Aşkar Z., Zafer C., İçli S. (2011). Effect of side chain substituents on the electron injection abilities of unsymmetrical perylene diimide dyes. Dyes Pigment..

[23] Ramanan C., Semigh A.L., Anthony J.E., Marks T.J., Wasielewski M.R. (2012). Competition between singlet fission and charge separation in solution-processed blend films of 6,13-bis(triisopropylsilylethynyl)-pentacene with sterically-encumbered perylene-3,4:9,10-bis(dicarboximide)s. J. Am. Chem. Soc..

[24] Shibano Y., Umeyama T., Matano Y., Imahori H. (2007). Electron-donating perylene tetracarboxylic acids for dye-sensitized solar cells. Org. Lett..

[25] Kozma E., Catellani M. (2013). Perylene diimides based materials for organic solar cells. Dyes Pigment..

[26] Tian H., Liu P.H., Zhu W., Gao E., Wu D.J., Cai S. (2000). Synthesis of novel multi-chromophoric soluble perylene derivatives and their photosensitizing properties with wide spectral response for SnO_2_ nanoporous electrode. J. Mater. Chem..

[27] Choi H., Paek S., Song J., Kim C., Cho N., Ko J. (2011). Synthesis of annulated thiophene perylene bisimide analogues: Their applications to bulk heterojunction organic solar cells. Chem. Commun..

[28] Huang C., Barlow S., Marder S.R. (2011). Perylene-3,4,9,10-tetracarboxylic acid diimides: Synthesis, physical properties, and use in organic electronics. J. Org. Chem..

[29] Wang H.Y., Peng B., Wei W. (2008). Solar cells based on perylene bisimide derivatives. Prog. Chem..

[30] Lu X., Guo Z., Sun C., Tian H., Zhu W. (2011). Helical Assembly induced by hydrogen bonding from chiral carboxylic acids based on perylene bisimides. J. Phys. Chem. B.

[31] Würthner F. (2004). Perylene bisimide dyes as versatile building blocks for functional supramolecular architectures. Chem. Commun..

[32] Wasielewski M.R. (2009). Self-assembly strategies for integrating light harvesting and charge separation in artificial photosynthetic systems. Acc. Chem. Res..

[33] Kaur B., Bhattacharya S.N., Henry D.J. (2013). Interpreting the near-infrared reflectance of a series of perylene pigments. Dyes Pigment..

[34] Langhals H., Kirner S. (2000). Novel fluorescent dyes by the extension of the core of perylenetetracarboxylic bisimides. Eur. J. Org. Chem..

[35] Liang Y., Wang H., Wang D., Liu H., Feng S. (2012). The synthesis, morphology and liquid-crystalline property of polysiloxane-modified perylene derivative. Dyes Pigment..

[36] Kaur B., Quazi N., Ivanov I., Bhattacharya S.N. (2012). Near-infrared reflective properties of perylene derivatives. Dyes Pigment..

[37] Cui Y., Wu Y., Liu Y., Yang G., Liu L., Fu H., Li Z., Wang S., Wang Z., Chen Y. (2013). PEGylated nanoparticles of diperylene bisimides with high efficiency of ^1^O_2_ generation. Dyes Pigment..

[38] Wang R., Shi Z., Zhang C., Zhang A., Chen J., Guo W., Sun Z. (2013). Facile synthesis and controllable bromination of asymmetrical intermediates of perylene monoanhydride/monoimide diester. Dyes Pigment..

[39] Luo M.-H., Chen K.-Y. (2013). Asymmetric perylene bisimide dyes with strong solvatofluorism. Dyes Pigment..

[40] Kang H., Jiang W., Wang Z. (2013). Construction of well-defined butadiynylene-linked perylene bisimide arrays via cross-coupling. Dyes Pigment..

[41] Daimon T., Nihei E. (2013). Fabrication of a poly(3-octylthiophene-2,5-diyl) electrochemiluminescence device assisted by perylene. Materials.

[42] Sharma G.D., Kurchania R., Ball R.J., Roy M.S., Mikroyannidis J.A. (2012). Effect of deoxycholic acid on the performance of liquid electrolyte dye-sensitized solar cells using a perylene monoimide derivative. Int. J. Photoenergy.

[43] Tsai H.Y., Chang C.W., Chen K.Y. (2014). 1,6- and 1,7-Regioisomers of asymmetric and symmetric perylene bisimides: synthesis, characterization and optical properties. Molecules.

[44] El-Daly S.A., Alamry K.A., Asiri A.M., Hussein M.A. (2012). Spectral characteristics and fluorescence quenching of *N*,*N*′-bis(4-pyridyl)-3,4:9,10-perylenebis(dicarboximide) (BPPD). J. Lumin..

[45] Tsai H.Y., Chen K.Y. (2014). Synthesis and optical properties of novel asymmetric perylene bisimides. J. Lumin..

[46] Naveenraj S., Raj M.R., Anandan S. (2012). Binding interaction between serum albumins and perylene-3,4,9,10-tetracarboxylate - A spectroscopic investigation. Dyes Pigment..

[47] Zhang L., Wang Y., Yu J., Zhang G., Cai X., Wu Y., Wang L. (2013). A colorimetric and fluorescent sensor based on PBIs for palladium detection. Tetrahedron Lett..

[48] Boobalan G., Imran P.M., Ramkumar S.G., Nagarajan S. (2014). Fabrication of luminescent perylene bisimide nanorods. J. Lumin..

[49] Ma Y.S., Wang C.H., Zhao Y.J., Yu Y., Han C.X., Qiu X.J., Shi Z. (2007). Perylene diimide dyes aggregates: Optical properties and packing behavior in solution and solid state. Supramol. Chem..

[50] Rajasingh P., Cohen R., Shirman E., Shimon L.J.W., Rybtchinski B. (2007). Selective bromination of perylene diimides under mild conditions. J. Org. Chem..

[51] Chen K.Y., Fang T.C., Chang M.J. (2011). Synthesis, photophysical and electrochemical properties of 1-aminoperylene bisimides. Dyes Pigment..

[52] Tsai H.Y., Chen K.Y. (2013). 1,7-Diaminoperylene bisimides: Synthesis, optical and electrochemical properties. Dyes Pigment..

[53] Ahrens M.J., Tauber M.J., Wasielewski M.R. (2006). Bis(*n*-octylamino)perylene-3,4:9,10-bis(dicarboximide)s and their radical cations: Synthesis, electrochemistry, and ENDOR spectroscopy. J. Org. Chem..

[54] Wang H., Kaiser T.E., Uemura S., Würthner F. (2008). Perylene bisimide J-aggregates with absorption maxima in the NIR. Chem. Commun..

[55] Zhao C., Zhang Y., Li R., Li X., Jiang J. (2007). Di(alkoxy)- and di(alkylthio)-substituted perylene-3,4;9,10-tetracarboxy diimides with tunable electrochemical and photophysical properties. J. Org. Chem..

[56] Zhang X., Pang S., Zhang Z., Ding X., Zhang S., He S., Zhan C. (2012). Facile synthesis of 1-bromo-7-alkoxyl perylene diimide dyes: Toward unsymmetrical functionalizations at the 1,7-positions. Tetrahedron Lett..

[57] Dhokale B., Gautam P., Misra R. (2012). Donor-acceptor perylenediimide-ferrocene conjugates: Synthesis, photophysical, and electrochemical properties. Tetrahedron Lett..

[58] Miasojedovasa A., Kazlauskasa K., Armonaitea G., Sivamuruganb V., Valiyaveettilb S., Grazuleviciusc J.V., Jursenasa S. (2012). Concentration effects on emission of bay-substituted perylene diimide derivatives in a polymer matrix. Dyes Pigment..

[59] Handa N.V., Mendoza K.D., Shirtcliff L.D. (2011). Syntheses and properties of 1,6 and 1,7 perylene diimides and tetracarboxylic dianhydrides. Org. Lett..

[60] Li Y., Tan L., Wang Z., Qian H., Shi Y., Hu W. (2008). Air-stable *n*-type semiconductor: Core-perfluoroalkylated perylene bisimides. Org. Lett..

[61] Chen K.Y., Chow T.J. (2010). 1,7-Dinitroperylene bisimides: Facile synthesis and characterization as *n*-type organic semiconductors. Tetrahedron Lett..

[62] Chen Z.J., Wang L.M., Zou G., Zhang L., Zhang G.J., Cai X.F., Teng M.S. (2012). Colorimetric and ratiometric luorescent chemosensor for fluoride ion based on perylene diimide derivatives. Dyes Pigment..

[63] Kong X., Gao J., Ma T., Wang M., Zhang A., Shi Z., Wei Y. (2012). Facile synthesis and replacement reactions of mono-substituteded perylene bisimide dyes. Dyes Pigment..

[64] Dubey R.K., Efimov A., Lemmetyinen H. (2011). 1,7- And 1,6-regioisomers of diphenoxy and dipyrrolidinyl substituted perylene diimides: Synthesis, separation, characterization, and comparison of electrochemical and optical properties. Chem. Mater..

[65] Würthner F., Stepanenko V., Chen Z., Saha-Möller C.R., Kocher N., Stalke D. (2004). Preparation and characterization of regioisomerically pure 1,7-disubstituted perylene bisimide dyes. J. Org. Chem..

[66] Lakowicz J.R. (1999). Principles of Fluorescence Spectroscopy.

[67] Chen Z., Baumeister U., Tschierske C., Würthner F. (2007). Effect of core twisting on self-assembly and optical properties of perylene bisimide dyes in solution and columnar liquid crystalline phases. Chem. Eur. J..

[68] Sheldrick G.M. (1997). SHELXS97, A Program for Automatic Solution of Crystal Structure.

[69] Sheldrick G.M. (1997). SHELX97, A Program for Crystal Structure Refinement.

